# Menstrual Cycle Dynamics and Their Impact on Psychotherapy: Insights From a Mixed‐Methods Study

**DOI:** 10.1002/cpp.70237

**Published:** 2026-02-10

**Authors:** Marie Anderlik, Jasmina Eifert, Manuela Gander, Anna Buchheim, Alexander Karabatsiakis

**Affiliations:** ^1^ Clinical Psychology II, Department of Psychology University of Innsbruck Innsbruck Tyrol Austria; ^2^ Department of Child and Adolescent Psychology Medical University of Innsbruck Innsbruck Tyrol Austria

**Keywords:** menstrual cycle, personalized medicine, psychotherapy, symptom exacerbations, treatment outcome

## Abstract

Despite substantial research on the physiological and psychological effects of the menstrual cycle (MC) on somatic and mental health, its impact on psychotherapy remains largely overlooked. Importantly, MC follows a recurring pattern of inflammatory activity. Emotional states, mood, cognitive functioning and sleep patterns fluctuate across the MC, affecting overall functioning and well‐being. Additionally, hormonal shifts across the cycle are linked to the exacerbation of psychiatric symptoms, particularly in premenopausal women who exhibit heightened sensitivity to normal changes in sex steroid levels. However, MC‐related hormonal fluctuations and inflammatory processes are rarely considered in psychotherapeutic settings. This exploratory mixed‐methods study examined how MC‐related fluctuations influence clients' psychotherapy experiences. In an online survey setting, a total of *N* = 425 female clients completed the Client Satisfaction Questionnaire‐8 (CSQ‐8) and the WHO Well‐Being Index (WHO‐5), alongside qualitative questions on symptom and therapy experiences. Most participants received cognitive‐behavioural (35.5%), psychodynamic (14.4%), systemic (9.4%) or humanistic (6.8%) therapy, while 33.9% were unsure of their therapy orientation, mostly in outpatient settings. Quantitative findings revealed reduced therapy satisfaction during premenstrual and perimenstrual phases compared with other phases. Qualitative data highlighted symptom exacerbation during these phases, negatively affecting emotional states, therapeutic participation, cognitive functioning and perceptions of therapy's necessity and effectiveness. Participants reported that the open discussion of the MC in therapy improved treatment outcomes. They advocated for individualized consideration of MC‐related factors in therapy planning. These findings underscore the need for more personalized clinical approaches that integrate MC‐related dynamics into psychotherapy to optimize treatment outcomes.

## Summary

- Psychotherapists should be aware that clients' emotional states, cognitive functioning and therapy satisfaction fluctuate across menstrual cycle phases.
- Premenstrual and perimenstrual phases are associated with lower therapy satisfaction and heightened symptom severity.
- Integrating menstrual cycle awareness into case formulation and therapy planning supports more personalized, phase‐sensitive interventions.
- Considering menstrual cycle dynamics in psychotherapy may offer clinically relevant insights and inform more personalized approaches to women's mental healthcare.

## 1 Introduction

The era of personalized medicine highlights the importance of tailored interventions that address individual variability. Similarly, recent psychotherapy research is moving towards personalized approaches and greater patient involvement in the therapeutic process (Cohen et al. 2021; Gerger et al. 2020). Although growing evidence suggests that the menstrual cycle (MC) may act as a cyclic intraindividual vulnerability factor, it remains an underexamined yet essential component in personalized approaches to women's health (Kuehner and Nayman 2021; Tauseef et al. 2024). For example, sensitivity to MC‐associated hormonal fluctuations of progesterone (P4) and estradiol (E2) varies greatly among individuals. Research indicates that up to 90% of women of reproductive age experience premenstrual symptoms, ranging from mild discomfort to premenstrual syndrome (PMS) or, depending on the severity of emotional and somatic impairment, premenstrual dysphoric disorder (PMDD; American Psychiatric Association 2013; Hantsoo, Rangaswamy, et al. 2022; Schoep et al. 2019). While the exact aetiology of MC‐related symptoms remains unclear, some potential underlying physiological mechanisms have been proposed, including elevated levels of inflammation and oxidative stress, aberrations in the allopregnanolone (ALLO) and GABAergic system and abnormal serotonergic activity interacting with the GABAergic system (Granda et al. 2021; Hantsoo and Epperson 2020; Schweizer‐Schubert et al. 2021). Women with severe premenstrual symptoms likely exhibit heightened sensitivity to normal hormonal fluctuations, with PMS representing an abnormal response to steroid surges (Schweizer‐Schubert et al. 2021). Similarly, it is suggested that an abnormal neural sensitivity to normal postovulatory ALLO surges triggers PMS/PMDD (Bixo et al. 2018; Hantsoo and Epperson 2020). Increased sensitivity to ALLO seems to interact with an underlying dysregulation in stress processing, causing premenstrual symptom expression (Bencker et al. 2024; Hamidovic et al. 2023).

Only recently have menstrual tracking applications begun generating a wealth of global data on common MC‐related symptoms such as dysmenorrhea, anxiety, mood swings and fatigue, with many women reporting disruptions to daily activities due to these symptoms (Cunningham et al. 2024; Hantsoo, Rangaswamy, et al. 2022; Pierson et al. 2021). Additionally, a growing body of research highlights the broader implications of cycle‐related sex steroid fluctuations for psychological well‐being and functioning, encompassing areas such as mood and emotions, learning and memory, cognition and sleep (Bencker et al. 2024; Bernal and Paolieri 2022; Derntl et al. 2024; Harrington et al. 2022; Hsu et al. 2021; Iqbal et al. 2024; Jeon and Baek 2023; Peters et al. 2024; Pletzer and Noachtar 2023).

Furthermore, fluctuating sex steroid levels are crucial in neuropsychiatric disorders, influencing both the biological networks underlying their pathogenesis and the severity and presentation of psychiatric symptoms (Iqbal et al. 2024; Kundakovic and Rocks 2022; Peters et al. 2024; Rubinow and Schmidt 2019). More precisely, MC‐related fluctuations are linked to the worsening of symptoms of an existing psychiatric condition, typically during the luteal phase preceding menstruation (Lin et al. 2024). The *International Society for Premenstrual Disorders* (ISPMD) classifies these premenstrual exacerbations (PMEs) as a variant of premenstrual disorders (Nevatte et al. 2013). Unlike PMS or PMDD, PMEs have received less attention in both research and clinical practice. However, like PMS and PMDD, PMEs are likely not caused by abnormal hormone levels but rather by an increased sensitivity to normal fluctuations in sex hormones and their metabolites (Lin et al. 2024; Peters et al. 2024). PMEs are of high clinical relevance as they create periods of heightened vulnerability, which may alter diagnostic evaluations, clinical risk analysis and treatment approaches (Lin et al. 2024; Mu et al. 2022; Peters et al. 2024). As a result, recent discussions advocate for PMEs to be formally recognized as a diagnostic subcategory (Peters et al. 2024).

Evidence suggests that PMEs can occur across multiple MC phases, including the mid‐luteal phase, perimenstrual phase and preovulatory phase, and have been recorded across various psychiatric conditions, including mood disorders, trauma‐related conditions, borderline personality disorder, anxiety disorders, psychotic disorders and obsessive‐compulsive disorder (Green and Graham 2022; Handy et al. 2022; Lin et al. 2024; Nillni et al. 2021; Nolan and Hughes 2022; Reilly et al. 2020; Wieczorek et al. 2023). Furthermore, research points to higher rates of female self‐harm, suicide attempts and suicide during the premenstrual and perimenstrual phases (Ross et al. 2023; Schmalenberger et al. 2024).

Despite the clear clinical relevance of MC‐related processes, their implications for psychotherapeutic research and practice have received extremely limited attention, even though MC‐related hormonal fluctuations may influence therapy outcomes by either enhancing or hindering therapeutic interventions (Fischer and Zilcha‐Mano 2022). For instance, in Dialectical Behavior Therapy (DBT), diary cards are often used to assess day‐by‐day fluctuations also related to PMS or PMDD‐related aspects; however, this information is clinically rather left unused. Also, in Cognitive Behavioural Therapy (CBT), the effectiveness for anxiety and fear‐related disorders appears to vary with hormone levels. Research shows that elevated progesterone levels during cognitive restructuring predict greater posttreatment reductions in behavioural avoidance in women with spider phobia (Li and Graham 2020). In contrast, higher estradiol levels are associated with reduced physiological arousal to conditioned stimuli and enhanced cognitive emotion regulation, suggesting that cognitive restructuring therapy may be more effective during estradiol‐rich phases (Graham et al. 2017). Higher estradiol levels are also positively associated with fear extinction recall (Garcia et al. 2018; Hsu et al. 2021). In women with severe posttraumatic stress disorder (PTSD), higher estradiol levels improve habituation during fear conditioning, reducing symptom severity and enhancing extinction‐based therapy outcomes (Sartin‐Tarm et al. 2020). Conversely, lower estradiol levels have been linked to slower treatment progress, increased fear and greater behavioural avoidance (Graham et al. 2018), highlighting estradiol's critical role in exposure therapy outcomes (Tang and Graham 2020).

Although PTSD and anxiety disorders have received considerable focus, other symptoms of mental disorders, such as eating problems, obsessive‐compulsive symptoms or interpersonal dysfunction are often neglected in this context. Moreover, PMEs are linked to poorer treatment responses, chronic illness courses and impaired functioning in bipolar and depressive disorders (Kuehner and Nayman 2021). On another note, therapy could potentially alleviate premenstrual symptoms by fostering improved emotion regulation and acceptance (Ishkova et al. 2024; Nayman et al. 2023). While evidence underscores the hormonal modulation of CBT‐related processes, the absence of comparable studies on other psychotherapeutic interventions, such as psychodynamic, humanistic or systemic approaches, represents a crucial and underexplored research gap. Addressing this imbalance requires extending MC‐related research beyond CBT paradigms to psychodynamic and integrative treatment models, which may be equally affected by cyclical variations in emotion and relationship dynamics. The impact of sex steroid fluctuations on psychological well‐being, functioning and psychiatric symptoms underscores the need for tailored psychotherapy approaches to optimize outcomes for hormone‐sensitive women. The goal of this mixed‐methods study was to assess the impact of MC‐related processes on psychotherapy outcomes and explore the client's perspective on the impact of the MC on psychotherapy. More specifically, we examined one hypothesis and three research questions. Our hypothesis stated that client satisfaction would be lower during the premenstrual/menstrual phases compared with other phases of the MC. In addition, we investigated the following research questions: (RQ1) Which MC‐related changes in symptoms, including PMEs, do clients report; (RQ2) in what ways do clients perceive the MC to influence their psychotherapy experience; (RQ3) how and why would clients consider the MC when planning, designing or scheduling psychotherapy sessions.

## 2 Methods

### 2.1 Study Design and Procedure

This study employed a mixed‐methods approach to investigate the influence of MC‐related processes on clients' psychotherapy experiences. The reporting of the mixed‐methods components follows the American Psychiatric Association (APA) JARS–Mixed (MMARS) guidelines (Levitt et al. 2018), to the extent possible given the exploratory design. Participants were recruited through online advertisements, mental health forums and university psychology networks. Inclusion criteria required participants to (i) have undergone psychotherapy and (ii) to have experienced at least one full MC while undergoing psychotherapy. All participants provided informed consent before participation, and responses were anonymous. Data collection took place between May 2023 and January 2024, using the *LimeSurvey* online survey tool. The questionnaire was available in English and German.

### 2.2 Sample and Descriptive Statistics

The sample included women who underwent psychotherapy while experiencing MCs (*N* = 425). In line with the exploratory aim of the study, no specific exclusion criteria were applied. Participants were included regardless of diagnosis, treatment history or hormonal status, to allow for a broad and ecologically valid understanding of MC‐related experiences across diverse clinical presentations. Of the participants, *n* = 419 provided additional qualitative data, with sample sizes varying by prompt. The questionnaire was completed in German (87.8%) and English (12.2%). The age range spanned from 18 to 58 years, with a mean age of M = 26.56 years (SD = 7.58 years). Regarding the highest level of education attained, the distribution was as follows: compulsory education (2.1%), high school diploma (40.2%), vocational training or apprenticeship (4.5%), bachelor's degree (34.6%), master's degree or diploma (17.2%) and PhD or doctorate (1.4%). In terms of current occupation, 67.3% of the participants were students, 25.9% were employed or working, 3.3% were unemployed, job seekers or receiving job training, and 3.5% fell into other categories. As for relationship status, 46.6% were single, 40.9% were in a relationship, 9.2% were married, 1.4% were divorced, and 1.9% reported other statuses. Participants were instructed to focus on their most recent therapy experience, with 52.2% currently undergoing psychotherapy. The median therapy duration was Mdn = 15 months (range: 1–176 months). The time since the last psychotherapeutic treatment was not assessed. Most participants received therapy in Austria (56.9%), followed by Germany (26.6%), Switzerland (0.9%) and other countries (15.5%). The majority attended outpatient therapy (88.5%), with session frequency varying from weekly (52%) to biweekly (31.5%), multiple times per week (3.5%) and less than biweekly (12%). Most participants received cognitive‐behavioural (35.5%), psychodynamic (14.4%), systemic (9.4%) or humanistic (6.8%) therapy, while 33.9% were unsure of their therapy orientation. Most participants had female therapists (86.8%; 12% male; 1.2% nonbinary/unsure). For an overview of the sample characteristics, see Table 1.

**TABLE 1:** Sample demographic characteristics.

Characteristic	Category	*n*	%
Age	M = 26.56, SD = 7.58, range = 18–58 years		
Language of questionnaire	German	373	87.8
	English	52	12.2
Education	Compulsory education	9	2.1
	High school diploma	171	40.2
	Vocational training	19	4.5
Highest educational level	Bachelor's degree	147	34.6
	Master's/diploma	73	17.2
	PhD/doctorate	6	1.4
Occupational status	Student	286	67.3
	Employed	110	25.9
	Unemployed/lob training	14	3.3
	Other	15	3.5
Relationship status	Single	198	46.6
	In a partnership	174	40.9
	Married	39	9.2
	Divorced	6	1.4
	Other	8	1.9

*Note: N* = 425; M = mean, SD = standard deviation.

#### 2.2.1 Psychotherapy‐Related Characteristics of the Study Sample (*N* = 425)

During therapy, some participants experienced events that could have influenced their hormonal status: 4.5% had a child, 17.6% underwent hormonal changes due to pregnancy, puberty or menopause, and 11.5% faced gynaecological conditions. Additionally, 28.5% reported changes in their use of antidepressants, benzodiazepines or antipsychotics, while 27.5% adjusted their hormonal contraceptive methods or hormone intake. In contrast, 42.4% reported no such changes. However, we found no significant correlation between these life events and client satisfaction scores.

### 2.3 Scales and Measures

The *WHO‐5 Well‐Being Index* (1998), a validated self‐report tool for subjective well‐being (SWB), ranges from 0 to 25, with scores below 13 indicating poor SWB and a need for depression screening. In this study, participants showed low SWB, with a mean score of 11.66 (SD = 4.96). The questionnaire demonstrated good reliability (Cronbach's *α* = 0.89). Client satisfaction was measured using the *Client Satisfaction Questionnaire‐8* (CSQ‐8), a standardized measure of client satisfaction with healthcare services (range: 8–32, higher scores indicating greater satisfaction). The CSQ‐8 was administered twice: once for psychotherapy sessions that occurred during the premenstrual/menstrual phase and once for sessions occurring outside those phases. Study results showed a mean score of M = 26.76 (SD = 4.88) for sessions during the premenstrual/menstrual phase, and M = 27.19 (SD = 4.73) for sessions outside those phases. Both had excellent reliability (Cronbach's *α* = 0.93).

To capture potential tendencies to downplay menstrual‐cycle experiences, three exploratory *Minimization and Denial* Items were included (‘I have always had a perfect menstrual cycle’, ‘There is nothing about my menstrual cycle that I have ever wanted to be different’, ‘Most women exaggerate the impact of their menstrual symptoms’). These items were inspired by the minimization and denial items of the *Childhood Trauma Questionnaire–Short Form* (Bernstein et al. 2003) and informed by clinical experience and observations. Items were rated on a 5‐point Likert scale (1 = *never true*; 5 = *very often true*). The scale had a mean score of 5.99 (SD = 2.30) with acceptable reliability (Cronbach's *α* = 0.63).

### 2.4 Qualitative Data Acquisition

Qualitative data were collected through open and semiopen questions exploring the relationship between psychiatric symptoms and the MC in psychotherapy. Participants responded to prompts such as ‘Describe the changes in symptoms of your psychiatric illness(es) throughout your menstrual cycle’ (if prior responses indicated such changes) and ‘What do you see as reasons for and/or against considering the MC when planning psychotherapy sessions?’. These items were self‐developed, as no validated measure captures clients' perceptions of the MC within psychotherapy. Open‐ended questions were used to elicit participants' subjective experiences, allowing greater qualitative depth and ecological validity than standardized symptom scales. To ensure comprehensive exploration, participants were also invited to provide final remarks on how their MC influenced their psychotherapy experiences.

### 2.5 Statistical Analysis

Data analysis was conducted using IBM SPSS Statistics 29.0 for Microsoft Windows. The significance level was set to *α* = 0.05 (one‐sided tests, *p* < 0.05 considered significant). Intercorrelations were assessed using Pearson's correlation. Internal consistency was evaluated via Cronbach's alpha. Outliers were defined using the interquartile range (IQR) method, identifying only mild outliers, leading to no participant exclusions. Hypothesis testing involved independent samples *t* tests. The normality of the dependent variable was assessed using the Shapiro–Wilk test, Q‐Q plots and histograms. Despite significant deviations from normality, the *t* test was applied due to its robustness in large samples. Levene's test was used to verify the equality of variances assumption.

### 2.6 Qualitative Data Analysis

Mayring's content analysis methodology was applied to evaluate responses to open‐ended questions, following a systematic, rule‐based and theory‐driven approach (Mayring 2022). This method, particularly effective for exploratory studies, involved paraphrasing, generalization and reduction to create categories based on structured research questions. A mixed deductive‐inductive approach was used, with some categories derived from existing literature (e.g., *RQ1_cat2: emotional and mood changes*), while others emerged directly from participant responses (e.g., *RQ3_cat4: concerns and cautions*). Data were collected in both German and English, with analysis conducted in English. Further, qualitative data were quantified to assess SEs across the MC.

## 3 Results

### 3.1 Self‐Reported MC Impact, MC Discussion Patterns and Symptom Fluctuations

**FIGURE 1:**
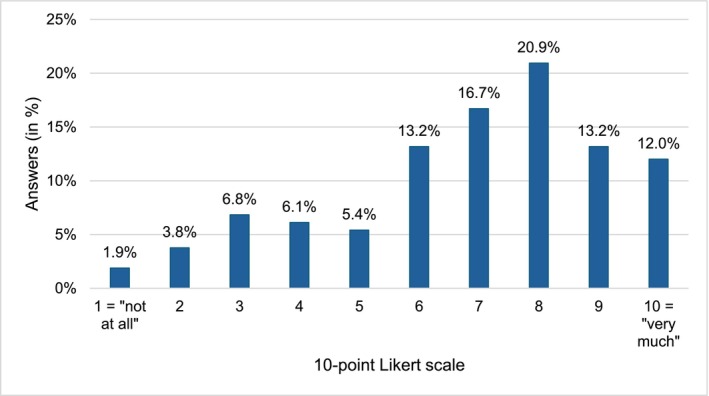
Self‐reported impact of the MC on well‐being.

On a single‐item scale (from 1 = “*not at all*” to 10 = “*very much*”) assessing the self‐reported impact of the MC on well‐being, the mean score was M = 6.84 (SD = 2.33), as shown in Figure 1.

**FIGURE 2:**
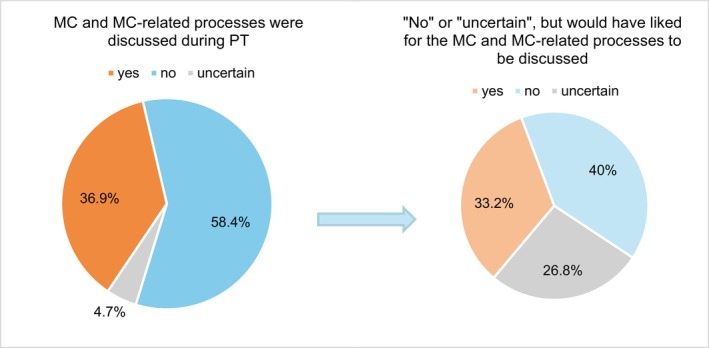
Menstruation cycle (MC)–related discussions and their need in psychotherapy treatment (PT).

Nearly half of the participants (48.9%) reported fluctuations in their psychiatric symptoms throughout the cycle, while 26.6% indicated no changes, and 24.5% were unsure. MC‐related symptoms prompted 5.4% of participants to seek therapy, and 7.1% had previously sought therapy specifically for MC‐related issues. Discussions about the MC or related bodily processes occurred in therapy for 36.9% of participants, while 58.4% reported no such discussions, and 4.7% were unsure. Among those who did not discuss the MC in therapy, 33.2% expressed interest in addressing it, 40% were not interested, and 26.8% were undecided (Figure 2).

**FIGURE 3:**
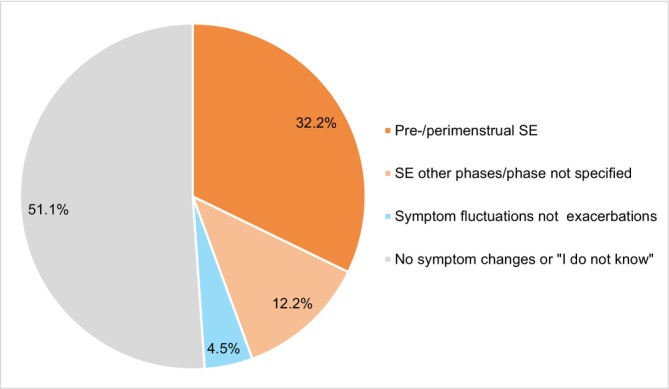
Symptom exacerbation according to cycle phase.

Among the study population, 44.4% reported experiencing SE of their psychiatric illness in response to the open‐ended questions (32.2% exacerbations during premenstrual and/or perimenstrual phases, 12.2% exacerbations in other phases/did not specify phase). Additionally, 4.5% noted symptom fluctuations without mentioning exacerbations, and the remaining 51.1% of participants did not mention symptom changes (also see Figure 3).

Intercorrelation analysis revealed several weak but significant relationships. Age was positively associated with therapy duration (*r* = 0.19, *p* < 0.01). *WHO‐5 well‐being* scores correlated with multiple factors: higher well‐being was linked to greater client satisfaction with therapy during both non‐menstrual (*r* = 0.17, *p* = 0.01) and premenstrual/menstrual phases (*r* = 0.22, *p* = 0.01), a lower perceived impact of the MC on well‐being (*r* = −0.19, *p* = 0.01), and higher scores on the minimization and denial scale (*r* = 0.24, *p* = 0.01). Additionally, the perceived impact of the MC on well‐being showed a moderate negative correlation with the minimization and denial scale (*r* = −0.31, *p* = 0.01). Lastly, a strong positive correlation was found between the two client satisfaction assessments (*r* = 0.93, *p* = 0.01).

### 3.2 Hypothesis: Lower Client Satisfaction During Premenstrual/Menstrual Phases

Clients reported lower satisfaction with therapy sessions during the premenstrual/menstrual phase compared with other phases. A paired samples *t* test revealed a significant difference in CSQ‐8 scores (*t*(424) = −4.94, *p* < 0.001), with lower satisfaction reported during the premenstrual/menstrual phase. The effect size was small (*d* = 0.24).

### 3.3 RQ1: MC‐Related Symptom Changes

The following categories encompass the symptom changes of their respective psychiatric illness(es) as reported by study participants: *RQ1_cat1: MC‐related symptom exacerbations* (symptom exacerbation mainly experienced in the premenstrual and perimenstrual phases; ‘I have OCD. My anxiety spikes a few days before my period. I get physical discomfort all around. That affects my stress which plays into my anxiety even more’, id 135), *RQ1_cat2: Emotional and mood changes* (reports of increased irritability, mood swings, depressive episodes, anxiety and feelings of vulnerability or sensitivity particularly during the premenstrual and perimenstrual phases; ‘I am more anxious and emotional unstable during my period. I tend to overthink and think less rational during my period’, id 269), *RQ1_cat3: Physiological symptoms* (descriptions of physical symptoms such as pain, fatigue or changes in appetite, and how these symptoms affect mental health; ‘I get physical discomfort all around. That affects my stress which plays into my anxiety even more’, id 135), *RQ1_cat4: Self‐perception and body image* (changes in self‐esteem, body image and self‐worth; ‘I tend to doubt myself way more and overall its way harder to fight the self‐sabotage thoughts than usual because im more tired so also more sensitive’, id 259), *RQ1_cat5: positive aspects and relief phases* (relief, increased positivity or improved mental health primarily in the time following menstruation and leading up to ovulation; ‘It would get worse before and during the menstrual cycle, but after it seemed to ease’, id 432).

### 3.4 RQ2: Perceived Influence of the MC on Psychotherapy

The following themes were identified to capture the common experiences: *RQ2_cat1: Impact of emotional states* (emotional fluctuations corresponding with the MC, impacting therapy through heightened emotional sensitivity, increased vulnerability and mood swings; ‘Sometimes I was more emotional or showed more emotions during the session (aka cried during the session)’, id 436), *RQ2_cat2: Effects on therapeutic participation and cognitive performance* (increased challenges in attending or participating during menstruation, mainly due to emotional states or physical discomfort/pain; ‘I have found treatment sessions more difficult during menstruation because I often feel my logical processing is fighting through the haze of period symptoms’, id 118), *RQ2_cat3: effects on perceived therapy necessity and effectiveness* (a stronger need for psychotherapy during the premenstrual and perimenstrual phases, along with perceived setbacks in their therapeutic progress; ‘About every other month I get extremely hopeless during my period session and I feel angry that‚ therapy doesn't work’, id 135), *RQ2_cat4: moderating role of communication and openness* (degree of openness and communication about the MC's impact on therapy influences the therapeutic relationship and outcomes; ‘After more than one year of therapy I can explain my feelings to my psychotherapist and that helps me a lot’, id 493), *RQ2_cat5: marginal or unclear MC impact* (MC's impact is either minimal, not strongly recalled, not closely monitored or overshadowed by more pressing concerns within the therapy context; ‘I would not say it impacted my therapy experience more or less than it impacts my life in general’, id 259).

### 3.5 RQ3: MC Consideration in Therapy Planning and Scheduling

**FIGURE 4:**
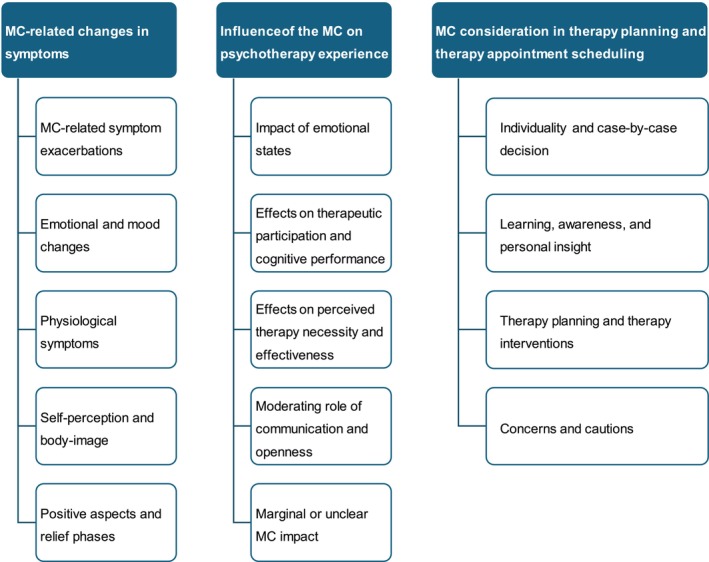
Qualitative data visualization.

The following key themes were identified: *RQ3_cat1: Individuality and case‐by‐case decision* (strong consensus on the necessity for a personalized approach in incorporating the MC into therapy; ‘I don't wish for the factor menstrual cycle to be considered for me, but if other women feel they need it considered, then it should be. Maybe therapists can ask this question during the first therapy session with a new female client’, id 114), *RQ3_cat2: Learning, awareness and personal insight* (discussing and considering the MC in therapy for gaining deeper personal insights and enhancing self‐awareness, and understanding the potential impact of the MC on their mental and emotional health; ‘I think that the therapist could be the person who not just explains why you feel different during the month, or that it is normal to feel lower during PMS, but also gives some techniques on how to deal with it’, id 252), *RQ3_cat3: MC consideration in therapy planning and therapy interventions* (varying resources and needs for psychotherapy across the MC; ‘I have different capacities (emotional, analytical) and needs in different phases. If my therapist knows that I have PMS, she can better understand my behavior’; id 862, translated from German), *RQ3_cat4: Concerns and cautions* (concerns about the proper consideration and potential negative implications; ‘PMS is used to dismiss or downplay women's emotional states as somehow less ‘real’ – I would be concerned that unless the practitioner was taking the distress very seriously, and there was a pre‐established relationship of trust and respect, it could feel dismissive or minimizing’, id 122; ‘I'm not female, I'm a trans man and that has a big impact on how I menstruate. There's a lot of shame and dysphoria attached to it, so I didn't want to address it much’, id 110; ‘Cultural factors come into play. The shame and embarrassment a lot of women can feel around their cycle could make the already difficult and vulnerable experience of trusting a psychotherapist, far more difficult’, id 122). For an overview of the results of the qualitative analysis, see Figure 4.

## 4 Discussion

### 4.1 Client Satisfaction

The reduced client satisfaction with therapy sessions during premenstrual and perimenstrual phases aligned with expectations, given the well‐documented increase in physiological discomfort (Hantsoo, Rangaswamy, et al. 2022; Schoep et al. 2019), heightened emotional sensitivity (Kimmig et al. 2022; Pierson et al. 2021; Pletzer and Noachtar 2023) and exacerbation of psychiatric symptoms (Green and Graham 2022; Handy et al. 2022; Nillni et al. 2021; Nolan and Hughes 2022; Reilly et al. 2020; Wieczorek et al. 2023). These findings suggest that clinicians might be mindful of cyclical fluctuations in mood and distress when interpreting session feedback. Implementing adaptive strategies, such as scheduling flexibility, symptom‐informed interventions or mindfulness‐based approaches, could potentially help mitigate the impact of MC‐related distress on therapeutic engagement. However, variations in therapy duration and the stage of treatment may also have influenced satisfaction rates (Lutz et al. 2021; Sembill et al. 2019). Future research should employ longitudinal designs to better control for therapy progress and should explore the impact of MC‐related nonattendance on outcomes.

### 4.2 Symptom Exacerbations

The symptom exacerbations reported in this study, particularly during the luteal phase, align with existing research (Green and Graham 2022; Handy et al. 2022; Nolan and Hughes 2022; Reilly et al. 2020; Wieczorek et al. 2023). Participants described worsened mood, increased depression and anxiety, heightened physical discomfort, pain and sleep disturbances during the premenstrual phase. Additionally, fluctuations in self‐esteem, body image and self‐efficacy were commonly noted. In contrast, many participants reported relief and improved mental health post‐menstruation or around ovulation, consistent with previously documented midcycle mood improvements (Pletzer and Noachtar 2023). These findings point to MC‐aware therapy that considers symptom variability across different phases. Cycle‐tracking might allow for anticipatory coping strategies during high‐symptom phases and leveraging midcycle improvements for therapeutic progress. Moreover, incorporating such cycle‐informed perspectives may also enhance the accuracy of psychotherapy outcome evaluations, as timing assessments without accounting for symptom fluctuations could risk underestimating or overestimating treatment effectiveness.

### 4.3 Impact of the MC on Psychotherapy Experience

One way the MC was perceived to influence psychotherapy was through widely reported emotional fluctuations, particularly in the premenstrual phase, aligning with existing research (Pletzer and Noachtar 2023; Sundström‐Poromaa 2018). Many participants struggled with therapy engagement during menstruation due to physical discomfort and emotional distress, consistent with evidence linking MC symptoms to impaired daily functioning (Hantsoo, Rangaswamy, et al. 2022; Schoep et al. 2019). Cognitive challenges, such as reduced concentration, were also highlighted, with participants attributing these to PMS‐related distractions. This underscores the importance of accounting for premenstrual complaints in future research on cognitive performance across the MC. While the premenstrual and perimenstrual phases increased therapy needs, they also were reported to be associated with perceived setbacks, occasionally causing doubts about therapy's effectiveness. At the same time, affective fluctuations may also hold therapeutic potential, for example by creating periods of heightened emotional accessibility in which latent affective processes or subjective meanings may become more salient and can be engaged with therapeutically when appropriately recognized by the therapist. Clinicians might consider helping clients distinguish temporary symptom‐driven fluctuations from actual treatment stagnation.

### 4.4 MC Communication and Personalized Care

Participants valued open discussions about the MC in therapy, which they experienced as strengthening the therapeutic relationship and enhancing therapy outcomes. They emphasized the importance of therapist‐client dialogue to address individual needs and differences in MC sensitivity, fostering self‐awareness and correcting misconceptions, underscoring the crucial role of psychoeducation in alleviating PMS‐related distress. The call for a more personalized integration of the MC into psychotherapy aligns with broader developments in personalized care and feedback‐informed treatment (Cohen et al. 2021; Huibers et al. 2021) and highlights the value of patient involvement (Gerger et al. 2020). Participants preferred having MC‐related discussions with female therapists, which is consistent with findings on patient‐therapist gender matching (Duong et al. 2025). While participants acknowledged the relevance of the MC, they also expressed concerns about overemphasis and stigmatization, advocating for a holistic, biopsychosocial approach that avoids reducing psychological issues to MC phases or reinforcing stereotypes. They emphasized the need for a nuanced understanding of MC effects in both research and clinical practice—one that supports individuals with premenstrual symptoms while avoiding generalized assumptions about all females (Eisenlohr‐Moul 2019). Future research could explore inclusive, tailored approaches that consider cultural, gender and stigma‐related factors, including the experiences of menstruating trans men.

### 4.5 Broader Therapeutic Implications of MC‐Aware Psychotherapy

Participants noted varying needs and resources across MC phases, suggesting that incorporating MC awareness into therapy planning and session timing could improve outcomes for some clients. Research supports the idea that hormonal fluctuations may influence intervention efficacy (Fischer and Zilcha‐Mano 2022; Rieder et al. 2020), particularly cognitive‐behavioural and exposure therapies (Glover 2020; Graham et al. 2017, 2018; Sartin‐Tarm et al. 2020). However, current knowledge is largely restricted to CBT, and little is known about how MC‐related variability might shape therapeutic processes in other modalities. Investigating psychodynamic therapy, family‐based approaches or mentalization‐based treatment could provide valuable insights, as these approaches target interpersonal functioning, affect regulation, mentalization and transference, domains that may themselves fluctuate across the cycle. Notably, early psychoanalytic authors also described cyclical variations in emotional and intrapsychic processes (Bálint 1937; Benedek and Rubenstein 1939b, 1939a), underscoring that the interplay between bodily cycles and psychological experience has long been recognized across therapeutic traditions.

A major strength of the study lies in its biological framing of psychotherapy. By conceptualizing the MC as a recurring physiological process that interacts with emotional states, cognition and psychiatric symptom expression, the study advances the view that psychotherapy should be responsive not only to psychological variables but also attuned to biological rhythms. The mixed‐methods design, combining large‐scale self‐report data with qualitative insights, enriches the understanding of how biological fluctuations manifest in the therapeutic setting.

### 4.6 Limitations

One limitation of the study is that it employs a retrospective design with no momentary or prospective assessments. Consequently, these aspects should be addressed in future studies. Generally, the retrospective and cross‐sectional design of this study limits its ability to capture within‐person MC dynamics, introducing social desirability effects and recall bias. Retrospective MC symptom reports are prone to false positives (Eisenlohr‐Moul et al. 2017; Schmalenberger et al. 2021), underscoring the need for daily symptom ratings to accurately evaluate PMEs and capture symptom variability across the cycle. A critical issue in MC‐related research is the ambiguity of terminology, which, combined with the participants' limited MC knowledge, may have influenced results (Schmalenberger et al. 2021). Although no formal attention or credibility checks were implemented, incomplete cases and a small number of qualitatively implausible or off‐topic responses were excluded prior to analysis to enhance data quality; nevertheless, inattentive responding cannot be fully ruled out. Additionally, uncontrolled confounding variables (e.g., hormonal contraceptive use) and potential sampling biases further restrict generalizability. Future research should use longitudinal designs with objective hormone and/or inflammatory measures and daily symptom tracking to improve accuracy. As an exploratory first step, the present study may help identify key methodological and conceptual considerations that should inform such longitudinal research, offering initial hypotheses and focal points for future investigations. Another important research direction concerns the question of how MC‐related intraindividual symptom fluctuations shape diagnostic impressions, as cyclical shifts in symptom severity may alter clinical presentations (Lin et al. 2024; Mu et al. 2022; Peters et al. 2024). Exploring different psychiatric disorders or disorder clusters could provide further insights—for instance, this study found that individuals with eating disorders were particularly affected by MC‐related physical symptoms, such as water retention and perceived weight gain. A follow‐up study could investigate neuroendocrine and inflammatory markers across the MC to identify subgroups with heightened sensitivity to hormonal fluctuations—potential endophenotypes—allowing for more personalized interventions. The findings also point to a broader educational gap, as clinically relevant MC‐related phenomena such as PME and PMDD remain insufficiently represented in psychotherapy training despite prior recommendations to include them more explicitly in professional curricula (Hantsoo, Sajid, et al. 2022; Kuehner and Nayman 2021). An additional question that warrants investigation is whether the MC phase at the initiation of psychotherapy influences early engagement, satisfaction trajectories or dropout risk.

## 5 Conclusion

This study offers preliminary insights into the influence of the MC on psychotherapy from the client's perspective. Findings indicate that therapy satisfaction fluctuates across MC phases, with lower satisfaction during the premenstrual/perimenstrual phases. Participants reported MC‐related symptom exacerbations, emotional and physiological changes, fluctuations in self‐perception and variations in therapy engagement and effectiveness. While many valued openness about the MC in therapy, concerns about stigmatization and oversimplification were also noted. Given the individualized impact of the MC, a personalized therapeutic approach may be more appropriate than standard treatment‐as‐usual. Expanding research, increasing awareness and integrating MC‐related training for mental health professionals could be important steps towards improving psychotherapy outcomes for female clients.

Overall, this study presents a first descriptive step in embedding biological dynamics into psychotherapeutic practice. It highlights the MC as both a source of vulnerability and an opportunity for individualized intervention. At the same time, methodological limitations, along with limitations in cycle‐phase assessment, measurement precision and sample diversity, underscore the need for longitudinal, biomarker‐informed and culturally inclusive research designs. Future studies integrating daily hormonal tracking and disorder‐specific analyses could provide the empirical foundation for a genuinely cycle‐informed psychotherapy while avoiding reductionist or stigmatizing interpretations.

## Author Contributions

Alexander Karabatsiakis (A.K.) developed the concept and design of the study. Marie Anderlik (M.A.) translated the concept and design into empirical practice under A.K.'s supervision as part of a Master's thesis. M.A. and A.K. evaluated the results and their interpretation, with critical input from Jasmina Eifert (J.E.), Manuela Gander (M.G.) and Anna Buchheim (A.B.). As first author, M.A. wrote the main manuscript, and A.K., as senior author, critically revised all stages. All coauthors approved the final version of the manuscript.

## Funding

The authors have nothing to report.

## Ethics Statement

The study protocol was approved by the Local Ethics Review Board at the Department of Psychology at Innsbruck University on 10 October 2022 under the registration number #90.

## Consent

All participants provided confirmation of consent before participating in the survey.

## Conflicts of Interest

The authors declare no conflicts of interest.

## Data Availability

The data that support the findings of this study are available on request from the corresponding author. The data are not publicly available due to privacy or ethical restrictions.
